# Pressure Distribution Inside Nucleons in a Tsallis-MIT Bag Model

**DOI:** 10.3390/e26030183

**Published:** 2024-02-22

**Authors:** Manuel A. Matías Astorga, Gerardo Herrera Corral

**Affiliations:** Physics Department, Centre for Research and Advanced Studies, CINVESTAV, P.O. Box 14 740, Mexico City 07360, Mexico

**Keywords:** hadrons, bag models, non-extensive statistics, Tsallis, QCD

## Abstract

We present a phenomenological framework based on the MIT bag model to estimate the pressure experienced by quarks and gluons inside nucleons. This is accomplished by implementing non-extensive Tsallis statistics for the two-component system. In this model of hadrons, the strong interaction generates correlations effectively described by the q-Tsallis parameter. The resulting hadron pressure exhibits general agreement with recent calculations derived from Lattice QCD. Additionally, we compared this pressure with data extracted from deep virtual Compton scattering experiments and gravitational form factor analyses. The extended bag model provides an alternative interpretation of bag pressure in terms of the q-Tsallis parameter. Consequently, the MIT bag model can be expressed without requiring the inclusion of the bag pressure parameter.

## 1. Introduction

The confinement of quark matter is a crucial area of investigation within Quantum Chromodynamics (QCD). Understanding the structure of hadrons is essential for unraveling the fundamental mechanism of strong interactions. Two main approaches have been developed to explore the physics of quark matter: on one side, Lattice QCD, which is a numerical technique that has yielded successful calculations, and on the other side, effective models. Both describe some physical aspects of hadrons, but not all. Over the years, various phenomenological descriptions of proton structures have been proposed, such as string models [1,2], which depict hadrons as oscillating strings; bag models [3,4], which consider quarks confined within a cavity; and valon models [5], which suggest that constituents of protons are clusters of partons called valons rather than valence quarks, among others.

Lattice QCD is a powerful computational approach used to study hadronic matter. However, it confronts some difficulties. It is computationally intensive for large lattice size and finer spacings, making some calculations potentially beyond the reach of current computing capabilities; high-density scenarios in QCD, such as heavy ion collisions and neutron star cores, remain a phenomenological challenge due to the so-called “sign problem,” among other issues. The sign problem is an important constraint in the theoretical characterization of physical states under varying conditions of temperature and pressure. Studies of the QCD phase diagram, where the chemical potential is nonzero, encounter difficulties arising from a non-Hermitian Dirac operator. 

Lattice QCD computations provide reliable results of hadron structures that contain heavy quarks and have recently been used as state-of-the-art calculations by the HAL-QCD collaboration [6,7]. The description obtained by HAL QCD of the final state strong interactions between proton–neutron and proton–hyperons systems was compared to the experimental results published by the ALICE Collaboration [8,9]. However, this groundwork does not need a detailed hadron structure modeling, and thus, no interacting components inside the involved baryons are considered. 

Effective models may be capable of a broader description in which both the hadron structure and its evolution in the phase diagram, when subject to extreme conditions of pressure and temperature, can be encompassed in a coherent picture. Here, we propose a phenomenological description based on the MIT bag model [10,11] and a non-extensive statistical approach. This could be referred to as the T-MIT bag model to abbreviate the merger of Tsallis non-extensive statistics and the MIT Bag Model [12]. To understand quark matter, it is crucial to have a model applicable to diverse scenarios and under various conditions, such as when a finite μ (chemical potential) is relevant to the system, as well as in cases when no chemical potential is involved. 

The extended bag model proposed here is centered on the hadron structure but may prove useful in understanding quark matter in regions of the QCD phase diagram with finite chemical potential, where Lattice QCD faces limitations.

The extended T-MIT bag model integrates different aspects of hadronic matter, allowing a more detailed study of processes in which a nonzero chemical potential enters. As an example, we explored its capabilities with a description of quark matter in a previous publication [12]. In that work, the implementation of nonadditive entropy of the quark and gluon systems as two probabilistic independent subsystems was used to obtain the QCD phase diagram. Now, we apply the formalism to obtain the pressure distribution inside nucleons, comparing models and recent estimates based on experimental observations. 

Tsallis statistics was introduced as a generalization of the Boltzmann–Gibbs statistics approach [13,14,15,16,17]. The Tsallis statistic has been widely used in high-energy physics. It was introduced for the first time to describe particle production in electron–positron collisions. The differential distributions of transverse momenta for charged hadrons with respect to the jet axis at several center-of-mass energies were successfully adjusted with a Tsallis function [18,19]. Tsallis distributions have also been used in proton–proton [20,21,22,23] and heavy ion [24,25] collisions to describe the experimental data. The observed success in describing the experimental data indicates that some underlying physics may be at work at a more fundamental level. 

Here, we incorporate the non-extensive Tsallis statistics in the MIT bag model to describe fermions and bosons as non-independent components of hadrons. In the new T-MIT bag model, one can estimate the total pressure distribution inside nucleons. We compared the resulting profile with the extracted pressure distribution of quarks published recently [26]. Indeed, the pressure distribution of the quarks inside the proton has been reported. The authors used experimental data from deep virtual Compton scattering (DVCS). It consists of probing the quark structure of the proton by scattering high-energy virtual photons radiated by electrons and the subsequent emission of a real photon. The photon in the final state allows the estimation of momentum transfer to the proton, which remains intact. The analysis relies on methods developed to extract information from Generalized Parton Distributions (GPD) and Compton Form Factors (CFFs). The study concludes that there is a repulsive pressure of the quarks near the center of the proton and a binding pressure at distances above 0.6 fm from the center. However, the extracted pressure distribution corresponds to one of the components of the proton, namely, the quarks.

On the experimental side, projects are now implementing techniques to gain more insight into the hadron structure [27], as well as the residual effects of strong nucleon-nucleon and nucleon–hyperon interactions [28,29]. The Electron Ion Collider (EIC) [30] planned for construction at Brookhaven National Laboratory will be dedicated to unravelling details of the strong interaction. It probes the transition between the perturbative and non-perturbative phenomena of QCD and the internal structure of protons and atomic nuclei. The EIC will reveal features of the sea of quark-antiquark pairs and key aspects of the gluon distribution. Effective models will continue to provide guidelines. The model proposed here offers a new approach to look at the structure of hadrons. We are not aware of similar advances aimed at estimating the pressure inside hadrons, although the fusion of the MIT bag model and Tsallis statistics has been performed previously [31,32]. In that reference, the authors analyzed the phase transitions in the QCD phase diagram to extract the stellar matter properties and observe the possible effects of non-additivity. The application of these ideas is, therefore, in different areas, and it will not be possible to compare the results. 

The goals of our work are threefold:

- Formulate the bag model considering the Fermi and Bose natures of the two components within the bag as correlated components.
- Replace the Bag Pressure with a function of the q-Tsallis parameter, which takes on a physical meaning in terms of the correlation between quarks and gluons. In other words, the proposed approach provides a reinterpretation of the main parameters in the bag models. Therefore, it does not introduce a new parameter but offers a new understanding of bag pressure.
- Apply the ideas to estimate a specific hadron property that is now under scrutiny, namely, the pressure distribution inside nucleons.

The remainder of this paper is organized as follows. In Section 2, a brief reminder of the conventional MIT bag model is presented. Here, we aim to identify aspects of the model that will be improved by introducing a non-extensive statistic. In Section 3, we explain the incorporation of Tsallis entropy into the MIT bag model to cast what we call the T-MIT bag model. Section 4 introduces structural characteristics of the proton that are useful for obtaining a better description of its pressure inside. In Section 5, we estimate the total pressure distribution. We comment on how one can make use of the quark pressure distribution, as reported in reference [26], to extract the pressure distribution that comes from the gluon. In Section 6, we formulate an expression that shows that the bag pressure can be replaced by the q-Tsallis parameter, and we explain what bag pressure can physically entail. In Section 7, we compare the obtained results with the pressure distribution reported from Lattice QCD models [33] and summarize them.

## 2. The MIT Bag Model

Bag models provide a natural mechanism of quark confinement in a Lorenz-co-variant form. They provide several hadron properties like charge radii, mass spectra, and magnetic moments [34] and offer a picture for recombination as part of the hadronization process that has been successful in describing spectra for all particle species in different colliding systems. Bag models have been used to obtain a heuristic understanding of the phase transition for quark matter and are popular for their simplicity and great phenomenological success. 

The MIT bag model treats quarks as free particles inside a cavity [10,11]. Confinement is then enforced with the requirement that the probability flow through the bag surface is zero, as well as with an external constant pressure on the bag that maintains quarks in a finite region of space. 

The model can be described with the Dirac equation inside the bag volume,

(1) $$\left(i\gamma^{\mu }\partial_{\mu }-m\right)\psi =0\;$$

and a boundary condition

(2) $$i\;n^{\mu }\gamma_{\mu }\psi ={\left.\psi \;\;\;\;\right|}_{r=R}$$

that implements confinement. This makes sure that the normal component of the vector current $J_{\mu }=\bar{\psi }\gamma^{\mu }\psi$ vanishes at the surface of the bag, i.e., the scalar product between the current and a four-dimensional vector $n^{\mu }$ that is perpendicular to the bag fades outside the cavity. 

In a scenario where quarks and gluons are massless and do not interact with each other inside the bag, ideal gases in thermal equilibrium with temperature *T* in volume *V* are considered. The total pressure of such a system is given by the sum of the partial components arising from the two ingredients:

(3) $$P_{total}=P_{Q}+P_{\bar{Q}}+P_{G}$$

The expression for these non-correlated components arising from quarks and gluons is obtained in standard textbooks [35],

(4) $$P_{Q}=\frac{g_{Q}}{3}\left[\frac{7\pi^{2}}{120}+\frac{1}{4}{\left(\frac{\mu }{T}\right)}^{2}+\frac{1}{8\pi^{2}}{\left(\frac{\mu }{T}\right)}^{4}\right]T^{4}\;g_{Q}=N_{s}N_{c}N_{f}=12$$

(5) $$P_{G}=g_{G}\frac{\pi^{2}}{90}T^{4}\;g_{G}=8\times 2=16$$

Here, we use Q and G as sub-indices to indicate the pressure due to Quarks and Gluons, respectively. The factors $g_{Q}$ and $g_{G}$ refer to the color degeneracy of quarks with 2 flavors (*f*), 3 colors (*c*), and 2 spin states (*s*). In a similar way for gluons with two polarizations and 8 color states. The number of antiquarks, and therefore the pressure they produce, is the same as that due to the quarks.

The MIT bag model describes hadrons as extended objects within a boundary subject to QCD vacuum pressure. This is considered by a crucial parameter, namely the bag pressure *B*:

(6) $$P_{system\;with\;a\;border}=P_{Q}+P_{\bar{Q}}+P_{G}-B$$

We will get back to this bag parameter later to obtain a better insight into the structure of hadrons.

In a grand canonical ensemble, we can get the expressions of entropy coming from each component,

(7) $$S_{Q}=g_{Q}\left[\frac{7\pi^{2}}{90}+\frac{1}{6}{\left(\frac{\mu }{T}\right)}^{2}\right]VT^{3}$$

(8) $$S_{G}=4g_{G}\left[\frac{\pi^{2}}{90}\right]VT^{3}$$

In the MIT bag model, one can add the pressures and entropies linearly, as in Equation (3), given the assumption of non-correlated independent subsystems. This idealized scenario is far from reality. In what follows, we will use a statistical formulation that handles non-additivity and provides a way to sum entropies for linked particle arrangements. We put together the pressure of the subsystems by considering the correlation that arises from the interdependence of the quark and gluon distributions. This can be done in a non-extensive approach to thermodynamics.

The bag pressure can be seen as an effective representation of the interaction between quarks mediated by the gluons. However, this interaction is complex. It involves not only a simple exchange of the strong interaction carrier but also self-interaction, gluon-gluon fusion processes, etc. An additional parameter that arises from the two components in the new statistical framework may be more realistic but will still be effective in the sense that it does not relate to a specific counterpart. 

## 3. Tsallis Entropy and the MIT Bag Model

A generalization of Boltzmann–Gibbs statistics was proposed by Tsallis [13,14,15,16,17]. While in traditional statistical mechanics, entropy is an extensive property, Tsallis statistics consider systems that may not satisfy its extensivity. In that formulation, a non-extensive entropy is defined as

(9) $$S_{q}=k\frac{1-\sum_{i=1}^{W}p_{i}}{q-1}\;\;q\in R$$

where q is a fixed index that characterizes a non-extensive system, W (∈ N) represents the number of microscopic configurations of the system, $p_{i}$ is the probability of configuration i. In Equation (9), k is a positive constant that turns out to be Boltzmann constant $k_{B}$ in conventional statistical mechanics.

The properties of this generalization are explained in reference [13]. As shown there, the entropy in the Boltzmann–Gibbs statistics is recovered from the new definition when q→1 .

An important aspect is the non-additivity relation of the Tsallis entropies. For two independent systems, *A* and *B*, a mixture of the two would be resumed by,

(10) $$\frac{S_{q}\left(A+B\right)}{k}=\frac{S_{q}\left(A\right)}{k}+\frac{S_{q}\left(B\right)}{k}+\left(1-q\right)\frac{S_{q}\left(A\right)}{k}\frac{S_{q}\left(B\right)}{k}$$

as formulated in reference [17] to underline the description of each system. For the specific application we are dealing with, *k* refers to the Boltzmann constant. 

The Legendre transformation of thermodynamics is *q*-invariant and can be used here.

Using the definition of $S_{q}$ several applications in high-energy physics have proved to be successful [36]. As mentioned before, the first indication of the great potential of this new statistic in high-energy processes was the description of particle production in electron–positron collisions [18,19]. Currently, it is a common way to characterize experimental data of particle production in a variety of high-energy reactions. 

Bag models are normally formulated in terms of non-interacting massless quarks and gluons; however, these two ingredients in hadrons do interact and are, therefore, correlated systems. Non-extensive Tsallis statistics give a prescription to treat such systems and measure the magnitude of the correlation.

The physical interpretation of the Tsallis *q* parameter depends on the specific system under consideration. It is often associated with correlations arising from long-range interactions. Systems with long-range interactions exhibit non-extensivity, and the level of correlation is represented by *q*, with higher values of *q* indicating stronger correlations. The connection between *q* and long-range interactions has been explored previously [37]. In that study, *q* is not considered to be a free parameter but a quantity depending on the physics involved. The analysis concludes that it may be related to the mean interparticle interaction length or screening length of the multiparticle system. More formal studies have been conducted [38] on the meaning of the *q* index that characterizes no extensivity and conjecture in a relationship with a system involving long-range interactions. 

We consider protons as a gas of quarks and gluons in this formulation of non-extensive systems with two components. Correlations between the two species are represented by the value of the Tsallis *q*-parameter. The non-extensive entropy for the hadron is then given by,

(11) $$S_{q}=S_{Q}+S_{G}+\left(1-q\right)S_{Q}S_{G}$$

where, $S_{Q}$ and $S_{G}$ are given in Equations (7) and (8). Henceforth, substituting and taking the degeneracy factors as given above, the entropy of the whole system can be written as,

(12) $$S_{q}=\left[\frac{74\pi^{2}}{45}+2{\left(\frac{\mu }{T}\right)}^{2}\right]VT^{3}+\frac{128\pi^{2}}{15}\left(1-q\right)\left[\frac{7\pi^{2}}{90}+\frac{1}{6}{\left(\frac{\mu }{T}\right)}^{2}\right]V^{2}T^{6}$$

We can then calculate the pressure using the Maxwell relations of thermodynamics by deriving $S_{q}$ with respect to V and then integrating the expression with respect to temperature T.

(13) $$P_{q}=\left[\frac{7}{4}g_{Q}+g_{G}\right]\frac{\pi^{2}}{90}T^{4}+\frac{1}{12}g_{Q}{\left[\frac{\mu }{T}\right]}^{2}T^{4}+\frac{8\pi^{2}}{90}g_{Q}g_{G}\left(1-q\right)\left[\frac{\pi^{2}}{90}\;\;+\frac{1}{30}{\left(\frac{\mu }{T}\right)}^{2}\right]VT^{7}+C\left(V,\mu ,q\right)$$

here, $C\left(V,\mu ,q\right)=\frac{1}{2\pi^{2}}\mu^{4}$. We recovered the total pressure for the conventional MIT bag (Equation (3)) by setting q=1.

By incorporating Tsallis entropy in the MIT bag model, we lost the independent contribution of the separate subsystems to the total pressure. We are not able anymore to isolate the pressure due to quarks from that due to the gluons. This makes sense, considering that protons are a complex, intertwined mixture of interacting quarks and gluons. Other approaches can detach fractions from each other. In the next section, we plot the total pressure and discuss how to use experimental extraction of quark pressure to gain some clues about the gluon contribution.

## 4. Proton Structure Characteristics

Information on the structural characteristics of some protons is very helpful. We incorporated some parameters of the proton structure to obtain a better insight into pressure.

Recently, results on gluon density have been published [39]. Analyzing the data, the gravitational density of the gluons was extracted. Measurements of the threshold J/Ψ photoproduction at the Jefferson Lab by experiment E12-16-007 [40] were used to derive the proton mass radius. It was found that the proton mass radius is smaller than its charge radius. Furthermore, this study yielded a scalar radius of 1 fm, which is larger than the charge radius. The results seem to indicate a structure consisting of regions inside the proton: an inner core followed by an extended volume of quarks that determines the charge radius and a confining envelope dominated by gluons that extend beyond the charged radius. 

The structure of nucleons was analyzed in a Bag Model, and the change in bag pressure with the radius of the proton was extracted. We incorporated this additional information. To estimate the pressure distribution, we employed a temperature profile dependent on the radius, as discussed in [41]. The authors assumed that the bag pressure could vary within a small range with volume and, consequently, it may change with radius. In [41], the relation between temperature and radius is given as $T=0.109R^{-3/4}\;\mathrm{GeV}$.

**Figure 1:**
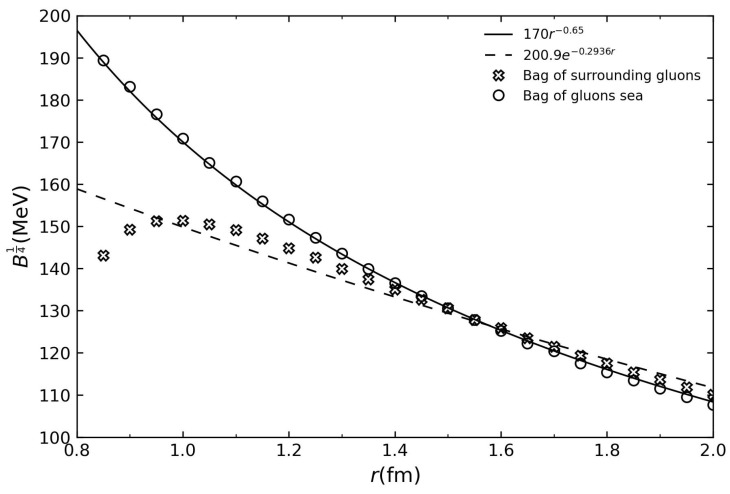
The bag pressure is examined under two different scenarios: the first one (cross dots) considers quarks surrounded by a sea of gluons, while the second (circle dots) considers quarks within the gluons’ sea. The adjusted curves were generated by considering the data of [41] for each case.

The structure in the bag model gives a pressure dependence on the radius as B14=0.17R−0.65 GeV corresponding to a scenario where the space inside is full of gluons and three quarks are swimming in the sea of gluons. A model in which quarks are enclosed by gluons produces a different dependence on the bag pressure. These are shown in Figure 1.

As a first approach, we use a bag pressure that is finite at the center of the hadron and vanishes at large distances. For this purpose, we have determined that a suitable approximation for the bag pressure is an exponential of the form: B14=200.9 e−0.2936r  MeV This expression aligns with the middle ground between the two curves shown in Figure 1. To derive this expression, we utilized data from the two models considered in [41]. In Figure 1, we compare the exponential bag pressure used with the scenarios presented in [41]. The distributions are similar in the range from 1.4 fermi onward. For large radii, the bag pressure tends to zero.

This aligns with the experimental indications, suggesting that an extended region populated by quarks is followed by a shell of gluons extending beyond the charge radius. 

## 5. Total Pressure Distribution and Gluons

**Figure 2:**
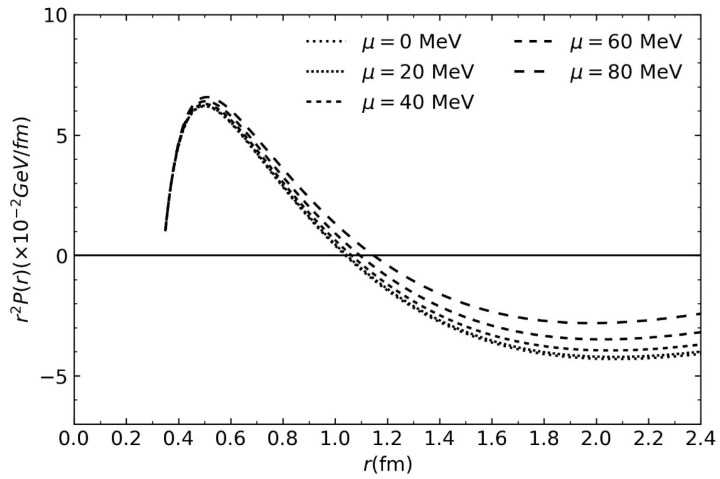
Radial pressure distribution in the proton versus radial distance from the center for different chemical potentials. The used Tsallis parameter was q=1.05.

The total pressure distribution obtained using Equation (13) is due to the quarks and gluons and can be seen in Figure 2. The pressure is shown as a function of the radius for several chemical potentials at a fixed *q* parameter. Please note that increasing the density of the particles at a given temperature hadrons will eventually result in deconfinement. This happens at densities of approximately 0.72fm3 or chemical potentials on the order of 430 MeV [35]. At higher temperatures, phase transition would be reached at lower densities.

The estimated distribution shows a repulsive pressure below 1 fermi and then a confining pressure above that distance from the center of the proton. 

The pressure distribution that results from the interactions of the quarks in the proton versus the radial distance from the center of the proton was obtained elsewhere [26] using experimental data. A strong repulsive pressure near the center of the proton was found to vanish at a radial distance of 0.6 fm. Beyond that, a binding pressure appears. In both cases, the extracted average peak pressure near the center is extremely high. 

In [26], the quark pressure distribution inside the proton is obtained by considering an isolated quark system without gluon interactions. In their analysis, they found the pressure distribution from Gravitational form factors (GFF) using the expression for

(14) $$d_{1}\left(t\right)=d_{1}\left(0\right){\left(1-\frac{t}{M^{2}}\right)}^{-\alpha }$$

which comes from the Gegenbauer expansion of the D-term (one of the GFF)

(15) $$D\left(z,t\right)=\left(1-z^{2}\right)\left[d_{1}\left(t\right)C_{1}^{3/2}\left(z\right)+\cdots \right]$$

**Figure 3:**
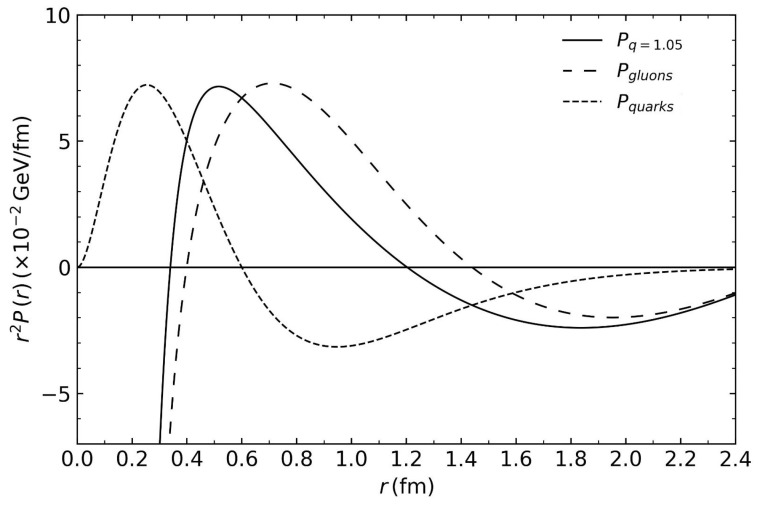
Extraction of the gluon pressure distribution from the central value in reference [26]. Chemical potential μ=100 MeV was used for the total pressure profile.

Here, $d_{1}\left(t\right)$ is related to the pressure distribution $p\left(r\right)$ via the spherical Bessel integral.

(16) $$d_{1}\left(t\right)\propto \int \frac{j_{0}\left(r\sqrt{-t}\right)}{2t}p\left(r\right)d^{3}r,$$

where $j_{0}$ is the first spherical Bessel function. One can find $p\left(r\right)$ in terms of $d_{1}\left(t\right)$. The pressure becomes.

(17) $$p\left(r\right)=-\frac{1}{k_{p}\pi^{2}}\int_{0}^{\infty }x^{4}j_{0}\left(rx\right)d_{1}\left(-x^{2}\right)dx=\;\;\;\;\frac{M^{6}d_{0}}{16\pi \left|M\right|k_{p}}e^{-\left|M\right|r}\left(-3+r\left|M\right|\right),$$

for α=3. Figure 3 shows the pressure with values of $k_{p}=55\;$and M=5 ($d_{0}$ has a constant value of $d_{0}=-2.04$).

The resulting pressure profile is shown in Figure 3. We used this quark pressure distribution to estimate the gluon component as a subtraction from the total pressure shown above. In doing so, we combined two different approaches; the resulting gluon pressure distribution is, therefore, only an estimated guess.

## 6. Physical Meaning of the *q* Parameter

In the bag model, quarks are confined within a region by the exchange of gluons, and the volume is characterized by pressure that prevents the quarks from escaping.

As indicated in reference [4], the Lagrangian density can be expressed as,

(18) $$L_{bag}=\left(L_{QCD}-B\right)\theta_{V}$$

where $\theta_{V}$ is the step function defining the interior of the bag containing quarks and gluons. It has a value of zero outside this region.

The model describes the interaction between quarks and gluons at small scales, reflecting the asymptotic freedom of the QCD. At larger scales, on the order of 1 fermi, quarks, and gluons become confined to color-neutral bound states. The Bag pressure, denoted by *B*, represents the energy density associated with vacuum fluctuations of the QCD fields inside the bag. 

In the analysis presented here, we did not assume the bag pressure to be constant throughout the region. We are aware of the intricate mechanism of confinement, which remains an active area of research.

Overall, the concept of bag pressure is somewhat artificial. It is introduced as a phenomenological parameter to describe confinement, and it is understood as the energy per unit volume of the vacuum fluctuations inside the bag. We can conceptualize the mechanism as a gluon sea-pushing quarks or as a sea of quarks and gluons interacting. In this way, the mechanism imparts structure to the hadron in accordance with the considerations made above. 

Now, we explore a more fundamental reason for the existence of bag pressure and find that introducing a correlation determined by the *q* parameter provides the possibility to eliminate *B* from the equations. By doing so, we aim to understand confinement without artificially introducing a bag pressure parameter. 

The Tsallis parameter *q* appears to encapsulate the physics involved in confinement, as it does the bag pressure. In other words, we can omit the bag pressure from this model by considering that at a given $q_{0}$ (initial Tsallis parameter), the bag pressure can be expressed for a general hadronic system.

(19) $$P_{q_{0}}\left(T,\mu \right)-B\left(r\right)\to P_{q}\left(T,\mu \right)$$

Here, q is the Tsallis parameter that describes the correlation, $q_{0}$ accounts for the estimated total pressure inside the nucleons above, with the given conditions (chemical potential, temperature, etc.). After extraction from the data, $q_{0}$ is fixed. In this way, we see that the Tsallis parameter can replicate the bag pressure in such a way that *B* is no longer needed. After some algebra, one can obtain the relationship between both the Tsallis parameter and the bag pressure as follows,

(20) $$q=q_{0}+\frac{B\left(r\right)}{\frac{256\pi^{2}}{15}\left[\frac{\pi^{2}}{90}+\frac{1}{30}{\left(\frac{\mu }{T}\right)}^{2}\right]VT^{7}},$$

the Tsallis parameter becomes dependent on the radius due to its reliance on r in the bag pressure. The possibility of developing a bag model of hadrons without the need for bag pressure will be explored in future work. Currently, we have noticed that the correlation arising from the quark and gluon systems as components of nucleons represents the bag pressure.

## 7. Results and Conclusions

**Figure 4:**
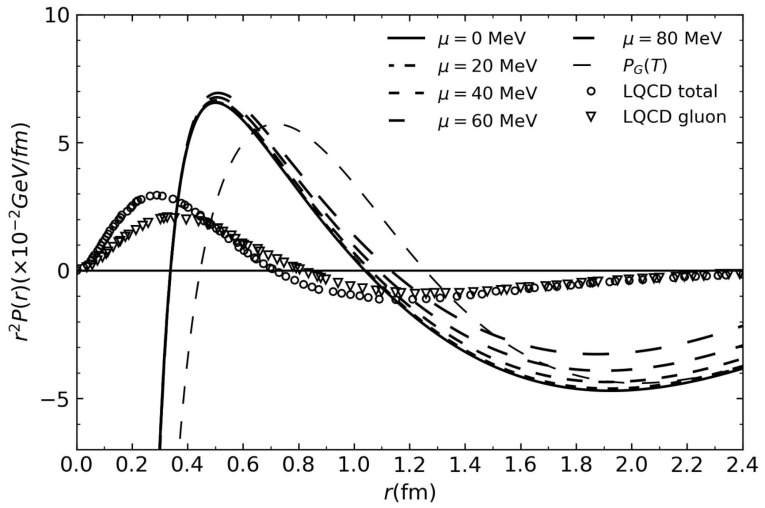
Lattice QCD results from reference [33] and those obtained with the modified MIT bag model.

Figure 4 shows the pressure distributions obtained with the T-MIT bag model together with the distributions of recent Lattice QCD calculations [33].

As mentioned above, the bag pressure and q-Tsallis parameter both represent effective aspects of the strong interaction mediated by quarks and gluons. We do not have a precise meaning for them but do have a specific relation between the *q* parameter and the actual phenomenology, as shown in Equation (20). We believe that the non-extensivity *q* value does have some connection with long-range interactions. 

To gain a better grasp of the physics behind this phenomenon, further analysis is needed. The understanding of experimental data, inclusive distributions in proton–proton, collective phenomena in heavy ion collisions, etc., is important to disentangle the reach and meaning of non-extensive statistics in nuclear matter. 

Here, we implemented Tsallis statistics to describe quarks and gluons as two correlated subsystems building a nucleon. We focused on the pressure inside the hadrons, but the idea can be extended to estimate the QCD phase diagram transitions, hadron masses, etc. [12,31,32]. 

The previous calculations presented here for comparison describe the general aspects of the pressure distribution. Those obtained recently from Lattice QCD and those using experimental data and Generalized Distribution Functions constitute the best estimate available; however, the conventional MIT bag model would predict a flat pressure distribution, as can be seen from Equations (3)–(6). In this regard, the T-MIT bag model represents a significant enhancement. 

References [42,43] introduced a similar approach to estimating pressure, energy, particle density, temperature, and chemical potential for a hadronic system in a non-extensive statistics bag model. In their study, the authors explored various scenarios and provided an estimation of the phase diagram depicting temperature versus chemical potential. Figures 7 and 8 in reference [42] illustrate the relationship between pressure and chemical potential for different values of the q-Tsallis parameter. Notably, there appears to be minimal difference among q = 1.10, 1.14, and q = 1.05 for chemical potentials below 0.1 GeV. These observations are consistent with our findings. Although the radial distribution of pressure is not investigated in their study, the overall pressure values are consistent with both the MIT bag model and our approach, as discussed in section VI of the reference.

Reference [43] offers a comprehensive examination of fractality, QCD and non-extensive statistics delving into the manifestation of scaling properties in Yang Mills field theory. This work forms the basis for the approach in [42].

In addition, an effective model is important considering its applicability to studying the phenomenology of processes where the chemical potential is nonzero.

It can be employed to model neutral matter in densely packed objects, such as neutron stars and other astrophysical bodies [31,32]. The residual QCD interaction may explain the effects observed as final-state strong interactions in nucleon–nucleon and nucleon–hyperon systems, but further quantitative studies are needed in this regard. The analysis here shows how pressure extends beyond the physical radius of the nucleons. How this may translate to residual effects is yet to be explored.

In summary, we have demonstrated that the Tsallis formulation of the MIT bag model is more realistic, as it effectively considers the correlation between the two components of hadrons, namely, quarks and gluons. This approach provides a better understanding of the bag pressure parameter, which can be substituted by a function of the Tsallis *q* parameter. Additionally, it can be applied to a specific description of hadrons, particularly for estimating the pressure inside the nucleons. 

## Data Availability

This manuscript has no associated data; henceforth, data will not be deposited. All data generated during the study are contained in this published article.
